# Achieving Protein Requirements Through Oral Feeding in Children with Congenital Heart Disease: A Narrative Review

**DOI:** 10.3390/children13091159

**Published:** 2026-08-28

**Authors:** Luciana B. Sutanto, Ray Wagiu Basrowi, Hilna Khairunisa Shalihat, Refani Alycia Kusuma, Nova Lidia Sitorus, Dessy Pratiwi

**Affiliations:** 1 Indonesia Nutrition Association, Jakarta 10430, Indonesia; 2 Danone Specialized Nutrition Indonesia, RDTX Place 9th Floor, South Jakarta 12940, Indonesia; nova.sitorus@danone.com (N.L.S.); dessy.pratiwi@danone.com (D.P.); 3 Occupational Medicine Division, Department of Community Medicine, Faculty of Medicine, Universitas Indonesia, Jakarta 10320, Indonesia; 4 Health Collaborative Center, Jakarta 14410, Indonesia; 5 Faculty of Medicine, Universitas Sumatera Utara, Medan 20155, Indonesia; hilna@rsham.co.id; 6 Adam Malik Hospital, Medan 20136, Indonesia; 7 Department of Nutrition, Faculty of Medicine, Universitas Negeri Semarang, Semarang 50229, Indonesia; refanialycia@mail.unnes.ac.id

**Keywords:** congenital heart disease, protein intake, oral feeding, pediatric nutrition, growth faltering, food fortification, oral nutritional supplementation, multidisciplinary care

## Abstract

**Highlights:**

**What are the main findings?**

- Children with congenital heart disease have increased protein requirements, while feeding fatigue, gastrointestinal dysfunction, and oral motor impairment frequently limit oral protein intake.
- Nutrient-dense feeding, food fortification, oral nutritional supplementation when appropriate, and multidisciplinary support may improve protein delivery and nutritional outcomes.

**What are the implications of the main findings?**

- Oral protein optimization should be individualized according to age, cardiac lesion, physiological status, and feeding capacity.
- High-quality CHD-specific prospective studies are needed to establish standardized, evidence-based recommendations for oral protein management.

**Abstract:**

**Background/Objectives**: Protein–energy malnutrition is common in children with congenital heart disease (CHD) due to increased metabolic demand and limited oral intake capacity. Achieving adequate protein intake in this population through oral feeding remains a critical yet underexplored challenge. This review aims to synthesize the currently available evidence on protein requirements, barriers to oral intake, and strategies for optimization of protein delivery in children with CHD. **Methods**: A narrative review was conducted using PubMed/MEDLINE, Scopus, and Web of Science. Relevant observational studies, randomized trials, guidelines, and recent reviews addressing nutritional management and oral feeding in pediatric CHD were included. Evidence was synthesized across metabolic, clinical, and practical domains. **Results**: Children with CHD exhibit increased protein requirements driven by hypermetabolism, inflammation, and perioperative stress, often reaching a requirement of 3–4 g/kg/day in high-risk settings. However, feeding limitations—including fatigue, gastrointestinal dysfunction, and oral motor impairment—restrict intake capacity and contribute to protein inadequacy in this population. Evidence supports the use of nutrient-dense feeding, optimized feeding frequency, food fortification, oral nutritional supplementation, and multidisciplinary care to improve protein delivery efficiency and nutritional status. **Conclusions**: Achieving adequate protein delivery through oral feeding requires a shift toward targeted, protein-focused strategies that address intake limitations. Early attention to protein adequacy may support nutritional recovery and growth, although its independent effects on postoperative and long-term outcomes remain uncertain. Further research is needed to establish standardized, evidence-based protocols tailored to this population.

## 1. Introduction

Congenital heart disease (CHD) remains the most common congenital anomaly worldwide, affecting approximately 0.8–1.2% of live births, although more than 85% of affected children now survive into adulthood due to advances in surgical and medical care [1,2]. Despite these improvements in survival, nutritional impairment—particularly protein–energy malnutrition—continues to represent a major and unresolved clinical challenge. Recent global analyses indicate that the prevalence of malnutrition in children with CHD remains alarmingly high, ranging from 15% to 64% globally, with pooled estimates demonstrating high rates of underweight (27.4%), stunting (24.4%), and wasting (24.8%) [3]. More recent studies have further suggested that up to 50% of children with CHD may experience acute or chronic malnutrition, significantly exceeding rates observed in healthy pediatric populations. This burden is particularly pronounced in low- and middle-income countries and is strongly associated with increased morbidity, prolonged hospitalization, and mortality [4]. Notably, preoperative malnutrition has been reported to be independently associated with adverse clinical outcomes, including prolonged mechanical ventilation, increased postoperative infection rates, extended intensive care unit (ICU) stays, and higher mortality [5].

The pathophysiology underlying malnutrition in CHD is complex and multifactorial, characterized by a dynamic interplay between increased metabolic demands, reduced nutrient intake, and impaired nutrient utilization. Children with CHD often have increased protein requirements due to hypermetabolism, chronic inflammation, and perioperative stress, while their oral intake is frequently limited by feeding difficulties and gastrointestinal dysfunction [6,7,8,9,10]. These factors collectively impair the ability of children with CHD to meet their elevated protein requirements, the satisfaction of which is essential for somatic growth, immune competence, and postoperative recovery [9,10].

Infants with CHD commonly experience feeding dysfunction characterized by reduced feeding endurance, poor suck–swallow coordination, and early satiety, which substantially reduce their feeding efficiency and oral protein intake [11,12]. Furthermore, longitudinal studies have highlighted that growth failure in early life is associated with impaired neurodevelopmental outcomes and reduced functional capacity later in life. Notably, current nutritional management strategies remain heavily skewed toward enteral and parenteral nutrition, particularly in perioperative settings, with comparatively limited emphasis on optimizing oral feeding [13,14].

Previous reviews and consensus statements have primarily focused on caloric adequacy, nutritional screening, and perioperative nutritional support, whereas strategies specifically aimed at achieving adequate protein intake through oral feeding have received comparatively limited attention [15]. This represents a critical gap, as oral feeding is not only the most physiological route of nutrient delivery but also essential for the development of oral motor skills, appetite regulation, and caregiver–child interactions [16]. Feeding difficulties in CHD patients are not solely volume-related but also reflect inefficiencies in nutrient density and protein delivery, underscoring the need for targeted strategies focusing on protein adequacy rather than caloric intake alone [17].

Given these considerations, there is a need to better define practical strategies for achieving protein requirements through oral feeding in children with CHD. This narrative review aims to synthesize the currently available evidence on the metabolic basis of increased protein needs, examine barriers to oral protein intake, and explore practical, evidence-based strategies to optimize protein delivery through oral feeding. By addressing this critical gap, the present review seeks to provide a practical clinical framework for optimizing oral protein intake and supporting nutritional management in children with CHD.

## 2. Materials and Methods

A targeted literature search of relevant English-language articles was conducted using major electronic databases, including PubMed/MEDLINE, Scopus, and Web of Science. The search aimed to identify studies addressing nutritional management, protein requirements, and oral feeding practices in children with congenital heart disease (CHD). The following keywords and their combinations were used: “congenital heart disease,” “protein intake,” “protein requirements,” “oral feeding,” “feeding difficulties,” “pediatric nutrition,” and “malnutrition.” The search was primarily limited to articles published between 2010 and 2026, with particular emphasis on studies from the last five years to capture the most recent evidence.

Throughout this review, the term “children” encompasses individuals from birth to 18 years of age with congenital heart disease. The review primarily focused on neonates, infants, and young children (0–5 years) with congenital heart disease, as this age group presents the highest prevalence of feeding difficulties, protein–energy malnutrition, and growth failure. Studies involving older children and adolescents (6–18 years) were included only when they provided clinically relevant evidence applicable across the pediatric age spectrum or addressed oral nutritional management relevant to younger children.

Eligible sources included observational studies (prospective and retrospective cohorts), randomized controlled trials, systematic reviews, clinical guidelines, and consensus statements relevant to nutrition in pediatric CHD. Articles focusing exclusively on enteral or parenteral nutrition without contextual relevance to oral feeding were selectively included only when they provided important comparative or mechanistic insights. Studies involving adult populations, non-cardiac conditions, or non-nutritional outcomes were excluded unless directly applicable to the conceptual framework of this review.

Given the narrative nature of this review, a formal Preferred Reporting Items for Systematic Reviews and Meta-Analyses (PRISMA) protocol was not adopted, consistent with previously published narrative reviews in high-impact journals. Articles were selected using a purposive approach based on their direct relevance to the objectives of the review, and studies were independently reviewed by two authors for relevance to the review objectives. Priority was given to systematic reviews, meta-analyses, randomized controlled trials, prospective cohort studies, international clinical practice guidelines, and high-quality observational studies with robust methodologies, adequate sample sizes, clearly defined outcomes, and direct applicability to oral nutritional management in pediatric CHD. Landmark publications were included if they provided foundational evidence that continues to inform current clinical practice. When CHD-specific evidence was unavailable, indirect evidence from pediatric critical care or other chronic pediatric conditions was incorporated cautiously and explicitly identified throughout the review. Studies reporting conflicting findings or uncertainties were also considered to provide a balanced interpretation of the current evidence.

**Figure 1:**
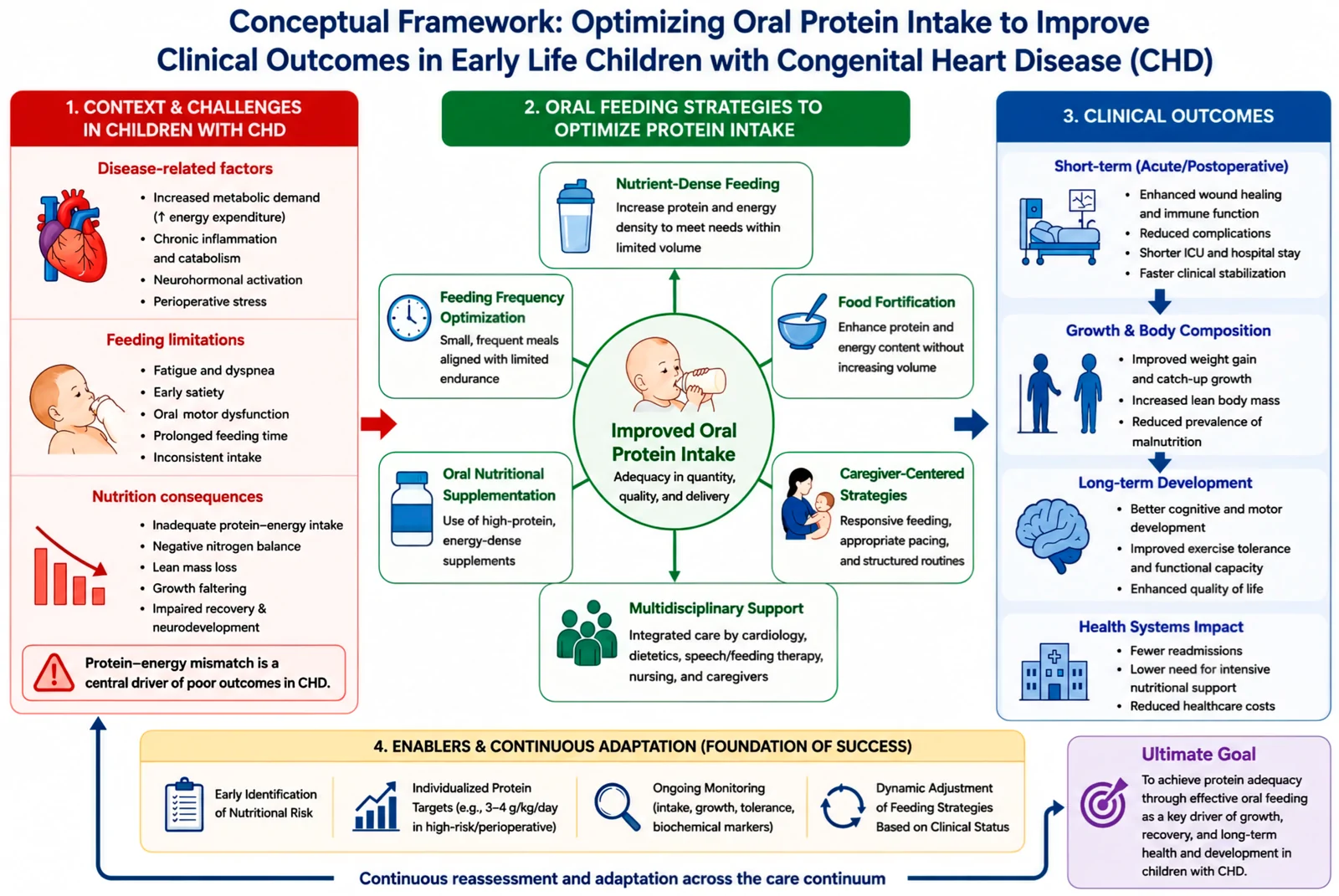
Conceptual framework for optimizing oral protein intake in children with congenital heart disease. Arrows indicate the proposed relationships among disease-related factors, feeding limitations, oral nutritional strategies, and clinical outcomes.

**Figure 2:**
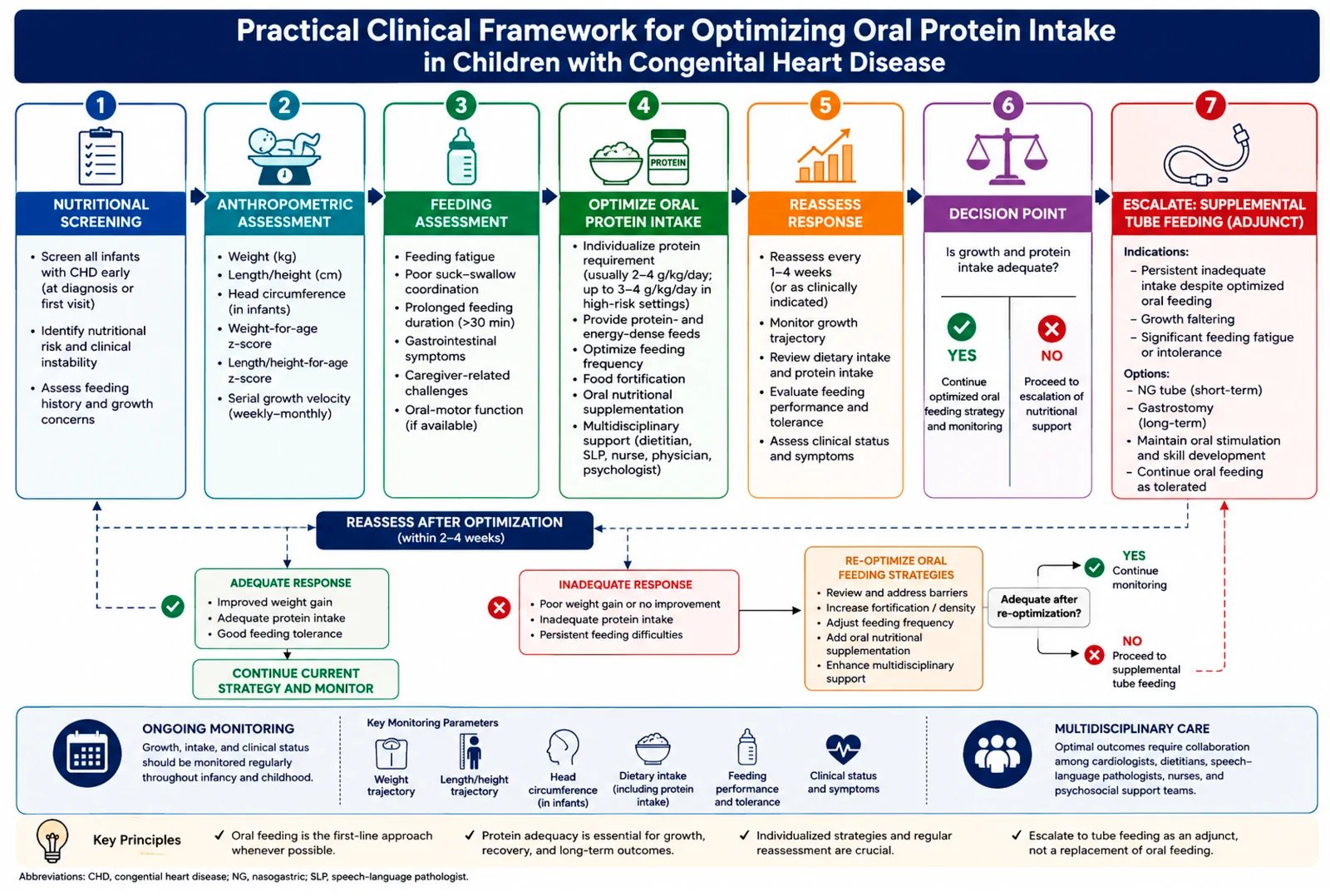
Practical clinical framework for optimizing oral protein intake in children with congenital heart disease. Dashed arrows indicate reassessment and decision pathways, while green and red indicators denote adequate and inadequate responses, respectively.

The conceptual framework presented in Figure 1 and Figure 2 was developed through an iterative narrative synthesis of the included literature. First, studies were grouped according to the primary domains addressed in this review; namely, metabolic and physiological determinants of increased protein requirements, barriers to achieving adequate oral protein intake, oral nutritional strategies, and clinical outcomes. Recurrent concepts and relationships consistently reported across high-quality studies and clinical guidelines were then identified and organized into an integrated conceptual model. The preliminary framework was reviewed and refined through iterative discussion among the authors, in order to ensure that it accurately reflected the current evidence and the intended scope of the review. The framework is intended to provide a visual synthesis of the evidence, rather than to represent a validated clinical model. The framework was not derived from a single study or guideline but represents the authors’ synthesis of the available evidence identified through the narrative review process.

This review was not intended to enable a detailed quantitative synthesis or meta-analysis; rather, evidence was synthesized narratively to provide a comprehensive and clinically meaningful overview. The review focused on the four following key domains: (1) physiological and metabolic determinants of increased protein requirements; (2) barriers to achieving adequate oral protein intake; (3) evidence-based oral nutritional strategies to optimize protein delivery; and (4) the clinical implications of protein optimization for growth, recovery, and long-term outcomes. Ethical approval was not required for this study, as it involved the analysis of previously published data and did not include human or animal subjects.

## 3. Protein and Energy Requirements in Children with Postoperative CHD

### 3.1. Metabolic Demand and Protein Turnover

Children with CHD present increased energy requirements due to persistent hypoxemia, shortness of breath, exhaustion, gastrointestinal disturbances, and limited heart function [2,18]. Furthermore, recurrent infection, surgical stress, and systemic inflammation may further intensify the catabolic state and elevate the metabolic demand in these patients.

Energy requirement is ideally assessed using indirect calorimetry as the gold standard. However, in clinical settings, predictive equations may be utilized as an alternative approach. Increased energy expenditure creates a challenge in terms of meeting the energy needs of children with CHD; for example, recent studies have demonstrated a 28–35% elevation in total daily energy expenditure in infants with CHD. In patients with heart failure (HF) and growth retardation, approximately 12% of initially ingested energy is lost through vomiting, alongside increased total daily energy expenditure and relatively low fecal energy loss [19]. Specifically, one study has reported that the energy requirements in children with heart failure can be increased by up to twice the values predicted using standard equations [20].

Elevated energy requirements in these patients are accompanied by protein metabolism disturbances, characterized by increased protein degradation, a predominance of catabolic pathways, and impaired anabolic signaling. This metabolic imbalance contributes to a negative nitrogen balance, progressive loss of lean body mass, and a suboptimal growth trajectory [21]. In the context of HF, inadequate protein intake further exacerbates skeletal muscle wasting and has been consistently associated with significantly increased mortality. Therefore, optimizing the provision of energy and protein to meet the higher metabolic demands during acute and chronic inflammation is crucial for enhancing clinical outcomes [22].

Current expert recommendations propose a risk-stratified approach to nutritional provision for children, indicating that those at low nutritional risk typically require around 90–100 kcal/kg/day and approximately 1.5 g/kg/day of protein, while children at moderate risk may need increased intake, in the range of 110–120 kcal/kg/day with about 2.5 g/kg/day of protein. In contrast, infants categorized as high-risk—especially those with hemodynamically significant CHF and coexisting malnutrition—frequently demonstrate elevated metabolic demands, with energy requirements rising to 120–150 kcal/kg/day and protein needs reaching up to 4 g/kg/day; in extreme cases, total caloric expenditure may approach up to three-fold the basal metabolic rate [2,23].

To provide an integrated overview of these interrelated factors, a conceptual framework is presented in Figure 1, illustrating the relationships between disease-related metabolic stress, feeding limitations, targeted oral nutritional strategies, and subsequent clinical outcomes.

### 3.2. Perioperative Stress and Recovery Needs

Surgery or another invasive procedure is the definitive treatment for CHD. About one-quarter to one-third of CHD cases in children are critical, requiring surgical or interventional care within the first year of life, whereas up to 49% of CHD cases require procedural care at least once in a lifetime [24]. Moreover, children with CHD represent approximately 30–40% of pediatric intensive care unit (PICU) admissions, primarily due to the need for specialized management of heart failure, arrhythmias, respiratory failure, and perioperative care [25].

Critical illness and injury triggers complex endocrine, immunologic, and metabolic responses aimed at maintaining homeostasis, including altered turnover of proteins, fats, and carbohydrates. Initially, glycogenolysis provides rapid energy to meet acute demands; however, depletion of glycogen stores shifts metabolism toward gluconeogenesis, utilizing amino acids to maintain vital organ, tissue repair, and wound-healing functions. This process is accompanied by increased synthesis of acute-phase proteins and leads to a hypercatabolic state characterized by impaired nutrient utilization, insulin resistance, and altered substrate metabolism. In the pediatric intensive care setting, these metabolic alterations are further compounded by feeding challenges, including gastrointestinal intolerance, fluid restrictions, mechanical ventilation, frequent interruptions, and hemodynamic instability, which hinder the ability to provide adequate nutritional support [25,26].

Energy requirements for critically ill children with CHD in the acute phase should not exceed the resting energy expenditure according to the Schofield equation, especially during the first 24–48 h, with minimal protein intake of 1.5 g/kg/day. Excessive calorie intake during the acute phase may lead to overfeeding, thus exacerbating metabolic stress, hyperglycemia, lipogenesis, and carbon dioxide overproduction, which can cause failure of weaning. Energy delivery can be gradually increased to 1.3–1.5 times the resting energy expenditure (REE). Patients may then move into the stable phase, which is marked by improved organ function and a partial resolution of the acute stress response despite ongoing endocrine changes. Subsequently, in the recovery phase—marked by restoration of hormonal balance and conversion to an anabolic state—higher energy provision (reaching up to 2 times the REE) is required to support tissue repair, catch-up growth, and lean body mass accretion [23,25]. This stepwise escalation of nutritional support is essential to optimize metabolic adaptation and meet the patient’s nutritional requirements.

### 3.3. Growth Failure, Catch-Up Growth, and Practical Growth Targets

Growth failure is a usual complication in children with CHD, which is characterized by inadequate growth, an inability to maintain growth, or a significantly decreased growth pattern, manifesting as stunting, underweight, and failure to thrive. Recent evidence has indicated that children with CHD show a marked decline in growth parameters, with a higher prevalence of growth failure when compared to non-CHD populations (46.3% vs. 7.5%) [27]. The prevalence rates were 24% for stunting, 29.3% for underweight, and 36.9% for failure to thrive in children with CHD. The severity of growth failure is related to the complexity of the underlying cardiac defect; in particular, children with more complex lesions are more vulnerable to growth deficits due to pathophysiological features including cyanosis, pulmonary hypertension, and reduced cardiac output, which disrupt endocrine regulation and inhibit musculoskeletal development. In addition, the cumulative effects of long-term drug therapy, repeated diagnostic procedures, multiple surgical interventions, and prolonged periods of intensive care create further challenges regarding nutritional status and growth potential [28].

Corrective surgery for CHD may also result in an optimized growth rate, referred to as “catch-up growth,” which can start as early as 3 months after corrective surgery. A cohort study involving 200 children with CHD who underwent surgery reported an ongoing reduction in the rate of weight growth disorder, from 44% preoperatively to 27% at 6 months, 9% at 1 year, and 3.5% at 2 years after surgery. Similarly, height and head circumference growth disorders presented reductions from 40.5% and 6.5% to 9.5% and 1.5%, respectively [29]. The recommended daily energy intake is approximately 175–180 kcal/kg, with protein intake at 6–8% of total calories to achieve catch-up growth and maintain adequate growth thereafter [23].

As growth recovery varies considerably among children with CHD, routine anthropometric monitoring is essential to evaluate nutritional responses and guide clinical management. Weight, length or height, and head circumference measurements should be interpreted according to age- and sex-specific growth standards, while growth velocity provides additional information on the efficacy of nutritional rehabilitation. In infants and young children, catch-up growth is generally considered when weight- or length-for-age z-scores improve over time and growth velocity exceeds the expected rate at a given age following correction of nutritional deficits [29]. Particular attention should be given to children with genetic syndromes commonly associated with CHD, such as Down, Turner, Noonan, and DiGeorge syndromes, for whom syndrome-specific growth charts may enable a more appropriate assessment than standard WHO growth charts [30,31]. Integrating serial anthropometric assessments with individualized nutritional targets allows for earlier identification of inadequate growth and supports timely adjustment of oral nutritional interventions before more intensive nutritional support becomes necessary. Practical recommendations for growth monitoring are summarized in Table 1.

## 4. Challenges in Achieving Protein Intake Through Oral Feeding

### 4.1. Feeding Fatigue and Cardiorespiratory Limitation

Children with CHD often experience early fatigue; this influences many aspects of their development, including feeding practice. Feeding fatigue is related to various feeding problems, such as those that arise from reduced feeding endurance and inadequate oral intake. Feeding problems are a common manifestation in CHD patients, with a reported prevalence of 42.9%, of which aspiration was reported in 32.9% [39]. These problems may result in a lack of exposure to oral feeding. One-third of families declared that they required longer feeding times and more frequent feeds. Consequently, many children with CHD fail to consume enough protein to meet their growth requirements, maintain normal metabolism and immune function, and recover effectively post-operation. A long-term study has reported that feeding disorders persisted in 11.5% of children with CHD for at least three years after open-heart surgery [40].

Another study reported prevalence rates of 34% and 16% for fatigue and severe fatigue in children with CHD, respectively [41]. Fatigue is closely related to underlying cardiorespiratory limitations, resulting in reduced cardiac output, hemodynamic instability, abnormal pulmonary perfusion, and early onset of anaerobic metabolism. Fatigue prior to feeding also interferes with the standard suck–swallow–breathe sequence, leading to increased aspiration risk and shortened feeding sessions. Furthermore, the child typically consumes more energy to feed than the nutrition they receive in the process. Consequently, shorter feeds, frequent feeds, and early supplemental enteral nutrition have been recommended to promote safe and sufficient protein intake and minimize cardiorespiratory stress [17].

### 4.2. Early Satiety and Gastrointestinal Factors

Early satiety and gastrointestinal dysfunction are major barriers to achieving adequate oral protein intake in children with CHD. In right-sided heart failure, hepatomegaly reduces gastric capacity while low cardiac output impairs gastrointestinal perfusion and delays gastric emptying, causing children to feel full after consuming only small amounts of food. Altered gastrointestinal hormone regulation—including reduced ghrelin and increased levels of satiety hormones such as peptide YY and glucagon-like peptide-1—may further suppress appetite and contribute to early satiety [25]. These alterations reduce feeding capacity and make it difficult for children with CHD to consume sufficient amounts of protein to meet their increased nutritional requirements.

Gastrointestinal dysfunction in CHD extends beyond impaired gastric motility. Gastroesophageal reflux disease is more prevalent in children with CHD than in healthy children, which may reduce voluntary oral intake as recurrent vomiting and feeding-associated discomfort create feeding aversion over time [42]. Reduced intestinal perfusion may also impair nutrient absorption through intestinal edema and mucosal injury. In children who have undergone Fontan palliation, protein-losing enteropathy (PLE) further aggravates nutritional compromise by increasing gastrointestinal protein losses while simultaneously reducing effective protein absorption, thereby substantially increasing protein requirements [43].

Inadequate protein intake may result from reduced intake, impaired absorption, or both, with consequences that extend well beyond impaired growth. Current guidelines recommend a protein intake of at least 2 g/kg/day in children with CHD, although achieving this target through oral feeding alone may be difficult in children with significant gastrointestinal dysfunction [23]. Therefore, clinical management should focus on improving feeding efficiency through small, frequent, protein-dense meals and timely treatment of reflux and gastrointestinal dysmotility. Oral feeding skills should be preserved whenever feasible, while oral nutritional supplementation should be introduced when dietary intake remains insufficient before considering escalation to supplemental enteral feeding.

### 4.3. Oral Motor Dysfunction

Oral motor dysfunction represents one of the most pervasive and clinically significant barriers to adequate protein intake through oral feeding in children with congenital heart disease (CHD). The pathophysiology of oral motor dysfunction is multifactorial, rooted in both the hemodynamic consequences of the cardiac lesion and postoperative sequelae. Tachypnea, chronic fatigue, and impaired oral motor coordination collectively limit effective feeding in infants with CHD, particularly those with cyanotic lesions or congestive heart failure [2]. Before food enters the gastrointestinal tract, it must first be safely swallowed; this is where many infants with CHD struggle most. Coordinated sucking, swallowing, and breathing is a neuromotor process that demands significant energy and respiratory control, which is often profoundly disrupted in this population. Infants who cannot sustain the rhythmic suck–swallow–breathe pattern are unable to consume adequate feeding volumes, directly compromising the intake of protein that is critical for anabolism, tissue repair, and growth [44].

Postoperative factors further compound pre-existing oral motor deficits. Vocal fold motion impairment (VFMI) arising from recurrent laryngeal nerve injury during cardiac surgery has been identified as a significant risk factor for aspiration and prolonged dysphagia. A retrospective study conducted at Lurie Children’s Hospital (2019–2024) involving 322 CHD patients, predominantly infants, found that VFMI substantially prolonged the time required to achieve full oral feeding. VFMI resolved in 35 (38.8%) patients within the median VFMI recovery time that was correlated with the time to dysphagia resolution (r  =  0.77, *p*  =  0.0001) [45]. A review has confirmed that vocal cord dysfunction diminishes airway protection during swallowing and heightens the risk of aspiration. Prolonged fasting periods surrounding surgical intervention and extended mechanical ventilation further suppress the development of oral feeding skills, creating a cycle of oral deprivation that exacerbates motor dysfunction and delays the reintroduction of protein-containing feeds [39].

Neurological and developmental impairments in children with CHD add another layer of complexity to oral motor dysfunction. Disruptions in feeding development among infants with CHD have been associated with delayed motor coordination and generalized neuromuscular weakness, which impair the postural stability necessary for safe and efficient oral feeding [44,46]. Infants who cannot maintain adequate flexion and stability of the head and neck are unable to generate sufficient sucking pressure or manage bolus transit effectively, resulting in prolonged feeding durations, fatigue-related early cessation and, ultimately, suboptimal protein consumption. Children with CHD represent a particularly high-risk group due to the heightened oropharyngeal coordination requirements for oral feeding at this developmental stage [46].

The clinical consequences of oral motor dysfunction extend beyond immediate inadequate protein intake, influencing long-term nutritional status and neurodevelopmental outcomes. A narrative review has highlighted that feeding disorders arising from sucking and swallowing dysfunction in CHD may trigger a cycle of inadequate intake, dysphagia, and nutritional deterioration [11]. Prolonged reliance on enteral tube feeding caused by the failure of oral feeding further attenuates oral motor skill development through a lack of exposure and oro-sensory deprivation [39]. Multidisciplinary interventions incorporating speech-language pathology, occupational therapy, and nutritional support are therefore essential to address oral motor dysfunction early, optimize protein delivery through oral routes where feasible, and prevent the downstream consequences of protein–energy malnutrition in this vulnerable population.

### 4.4. Behavioral and Caregiver-Related Factors

Family caregivers of children with CHD need to better understand the disease and develop skills to help with home care after hospital discharge. Children with CHD require specialized care, aimed at maintaining cardiac function and addressing challenges related to the complexity of the treatment [47]. Caring for a child with CHD is so significant that it can change the family’s routine, directly affecting the caregiver’s personal and professional life. Many parents experience difficulties in managing the child’s illness and their caregiving role, due to the need to reschedule activities, integrate the child’s needs into family life, and implement strategies to manage the condition [48].

Caregivers also have a significant role in establishing successful oral delivery, by shaping adaptive feeding behaviors and establishing healthy eating habits. This is crucial for children with CHD, who frequently experience feeding difficulties such as oral motor dysfunction, early satiety, gastrointestinal disturbances, and feeding fatigue—difficulties that have been associated with increased caregiver distress. Parents of children with CHD and feeding difficulties have reported a significantly more negative mood-state and significantly longer time associated with feeding, when compared to parents of children in the other two groups [48].

As evidenced by elevated levels of anxiety, depression, and psychological distress, a caregiver’s incorrect understanding of the child’s condition may lead to maladaptive feeding behaviors such as forced feeding, excessive distraction feeding, or underfeeding. Such an anxiety-based feeding environment has been shown to trigger feeding refusal in children. Aside from the caregiver’s mental state, socio-economic factors also affect oral feeding outcomes; for example, in a cross-sectional study, Ruan et al. (2024) found that parental unemployment and low family income may be associated with underweight, while parental employment and greater financial stability are more likely linked to nutritionally adequate feeding behaviors [49].

Family caregivers have reported a perceived lack of accessible feeding information for children with CHD [50], and have primarily turned to social networks and parent groups of children with the same condition in the search for such information. Feeding infants and children with CHD represents a significant challenge for caregivers, and close collaboration with professionals is necessary to ensure adequate caloric and nutritional intake for appropriate growth and weight gain [11].

Facilitating the mitigation of feeding difficulties through structured caregiver education and guidance regarding feeding practices can reduce caregiver-related emotional distress and frustration. Importantly, this should include tailored feeding practices adjusted to the child’s clinical condition, socio-economic context, cultural background, and the caregiver’s capacity. This approach can be expected to not only improve the adequacy of oral nutritional intake, but also to contribute to better clinical recovery and growth outcomes [51].

## 5. Oral Feeding Strategies to Achieve Protein Requirements

### 5.1. Increasing Nutrient Density

#### 5.1.1. Energy- and Protein-Dense Feeding Approaches

Energy- and protein-dense feeding may be a useful oral strategy for improving protein delivery in children with CHD, particularly when the feeding volume is limited. Rather than increasing the feeding volume, increasing the nutrient density enables greater protein and energy delivery within volumes that children can tolerate [23]. Available evidence suggests that nutrient-dense feeding may improve protein adequacy and weight gain, although evidence regarding postoperative recovery remains limited and heterogeneous [52,53]. Metabolic evidence supports the role of protein-enriched intake in improving nitrogen balance and preserving lean body mass during catabolic stress [2,54].

Protein-dense oral feeding may help address the increased protein demands associated with postoperative catabolism and inflammation. During the postoperative period, amino acids are preferentially utilized for gluconeogenesis and acute-phase protein synthesis, leading to depletion of skeletal muscle if intake is inadequate [55]. Increasing protein density may increase the availability of amino acids for anabolic processes—including muscle protein synthesis and tissue repair—while minimizing reliance on endogenous protein stores [56]. Adequate protein provision is also associated with improved immune function and modulation of the inflammatory response, both of which contribute to improvements in postoperative recovery in children with CHD [25,57].

Potential benefits of nutrient-dense feeding may extend beyond short-term nutritional recovery. Early nutritional adequacy has been associated with more favorable growth trajectories in children with CHD [2,58]. These findings highlight that optimizing the nutrient composition—particularly protein density—rather than feeding volume alone is central to achieving sustained nutritional adequacy [59].

#### 5.1.2. Food Fortification and Dietary Modification

Food fortification may be a useful oral strategy for addressing protein–energy deficits in children with CHD, particularly when oral intake is limited [60]. By increasing the protein and energy density of oral intake through fortified milk, growth formulas, or enrichment of habitual foods, clinicians can deliver substantially greater amounts of indispensable amino acids and energy within the limited volumes that these children can tolerate. This approach may be particularly relevant when feeding capacity is constrained by limited tolerable volumes [46]. Available evidence suggests that energy- and protein-enriched feeding regimens may improve weight gain and protein adequacy, although findings vary across populations and intervention setting [61].

Food fortification may improve the efficiency of nutrient delivery by increasing the amount of protein and energy provided within each feeding session [62]. During the postoperative period, higher protein availability supports tissue repair, muscle protein synthesis, and recovery while reducing reliance on endogenous protein stores [63,64]. Studies in pediatric critical illness contexts have demonstrated that higher protein intake is associated with improved nitrogen balance and preservation of fat-free mass, particularly when delivered early during the recovery phase [65]. Adequate energy intake also exerts a protein-sparing effect by reducing protein oxidation, allowing amino acids to be preferentially utilized for anabolic processes rather than energy production. This protein-sparing effect is especially relevant in CHD patients, in which a prolonged negative energy balance exacerbates lean mass loss and delays recovery [66].

Food fortification may facilitate more consistent nutrient delivery when feeding volume is limited in children with CHD [2,58]. In perioperative settings, optimized protein and energy intake has also been associated with faster clinical stabilization and fewer complications, highlighting nutrition as a modifiable determinant of recovery [15]. From an implementation perspective, food fortification is feasible, cost-effective, and adaptable across healthcare settings. It can be standardized through hospital protocols (e.g., predefined fortification targets) and continued at home with caregiver guidance, ensuring continuity of care across settings [67].

These findings support consideration of food fortification as one practical oral strategy when feeding volume is limited in children with CHD. Prioritizing nutrient density over feeding volume may facilitate achievement of protein requirements, particularly when feeding volume is limited, although evidence for effects on recovery and long-term outcomes remains limited.

#### 5.1.3. Oral Nutritional Supplementation

Oral nutritional supplementation (ONS) may be considered as a practical, non-invasive strategy to bridge the gap between dietary intake and increased protein–energy requirements in children with CHD, particularly when oral intake is insufficient [2,68]. Modern pediatric ONS formulations deliver high-quality protein (often enriched in essential amino acids such as leucine), energy-dense macronutrients, and a complete micronutrient profile within small volumes, thereby aligning with the limited feeding capacity typical of this population [68]. By increasing the nutrient density without substantially increasing the feeding volume, ONS may help increase protein and energy intake during periods of increased nutritional demand [56,68]. Evidence from pediatric critical care studies has indicated that higher protein intake is associated with improved nitrogen balance and preservation of lean body mass during recovery [69].

In pediatric populations at risk of undernutrition, ONS has been associated with increased nutrient intake and improvements in selected growth outcomes [70]. However, the extent to which these findings apply specifically to children with CHD remains uncertain. Although CHD-specific trials remain limited, the available evidence indicates similar benefits in children with cardiac conditions; especially among those with persistent feeding difficulties or poor appetite, in whom ONS may increase nutrient intake without prolonging feeding duration or increasing feeding-related fatigue [46,71].

When ONS is used, it may be incorporated into an individualized oral feeding plan rather than used as a meal replacement. Standardized protocols defining the timing, target volumes, and protein goals have been associated with more consistent achievement of protein requirements in clinical practice. ONS is generally well tolerated, with minimal gastrointestinal side effects when introduced gradually and tailored to individual tolerance; however, periodic monitoring remains important to ensure an appropriate nutrient balance is achieved [72,73]. Given its practicality and ability to deliver concentrated nutrients within limited volumes, ONS may be considered an integral component of oral feeding strategies for children who are unable to meet their protein requirements through diet alone.

### 5.2. Feeding Frequency Optimization

Optimizing feeding frequency through smaller, more frequent meals may help facilitate cumulative protein and energy intake in children with CHD who have limited feeding endurance [17]. Rather than increasing the feeding volume in a single feeding session, this approach provides incremental nutrient delivery while reducing fatigue associated with prolonged feeding sessions [74,75]. Structured feeding schedules are associated with improved total daily intake and greater achievement of protein targets, particularly during the early postoperative period when feeding tolerance is lowest. Distributing intake across multiple feeding episodes may facilitate feeding efficiency and reduce the metabolic burden associated with prolonged feeding sessions [53].

The physiological benefits of this strategy are supported by evidence demonstrating that prolonged feeding increases energy expenditure in infants with CHD due to sustained cardiorespiratory effort [76,77]. Indirect calorimetry-based studies suggest that shorter feeding sessions with adequate recovery intervals may reduce feeding-related metabolic demands while preserving intake capacity [2]. More frequent feeding helps to maintain more stable glucose and amino acid availability, supporting continuous protein synthesis and limiting catabolic fluctuations. Maintaining a positive protein balance is particularly important during the postoperative period, in order to support tissue repair and immune function [78].

Feeding frequency optimization may improve the efficiency of oral protein delivery without increasing the feeding burden [79]. As a low-cost and scalable intervention, this approach can be implemented in both hospital and home settings, making it particularly relevant in resource-limited environments [80]. These findings suggest that structured, frequent feeding schedules may be considered as part of individualized nutritional management to improve the achievement of protein requirements in children with CHD.

### 5.3. Supplemental Tube Feeding

Although oral feeding remains the preferred route for nutritional support in children with CHD, supplemental tube feeding should be considered when individualized oral feeding strategies fail to achieve adequate nutritional intake or support satisfactory growth. Rather than replacing oral feeding, tube feeding should be used as an adjunct to optimize protein and energy delivery while preserving oral feeding skills whenever feasible [46].

Nasogastric (NG) tube feeding is commonly used for short-term nutritional support because it is less invasive and allows for the continuation of oral feeding alongside enteral supplementation [81]. Night-time NG feeding combined with daytime oral feeding may be particularly beneficial for children who experience feeding fatigue, prolonged feeding duration, or increased metabolic demands [82]. Gastrostomy should be considered when prolonged enteral support is anticipated or when repeated NG tube placement is poorly tolerated, although the decision should be individualized according to the patient’s nutritional status, cardiac condition, and expected duration of support [83].

Escalation from oral feeding alone to supplemental tube feeding should be considered in children with persistent growth faltering, inadequate oral intake despite optimized dietary modification and multidisciplinary intervention, or when feeding results in significant fatigue, respiratory distress, or hemodynamic instability. Even when tube feeding is required, opportunities for oral stimulation and developmentally appropriate oral feeding should be maintained to support oral motor function and facilitate the transition back to full oral feeding. Early involvement of a multidisciplinary team, including dietitians, speech and language therapists, occupational therapists, and pediatric cardiologists, is important for individualized nutritional management and may help reduce long-term feeding dependence [23,84,85].

### 5.4. Multidisciplinary Support

The complexity of nutritional management in children with CHD necessitates a structured, multidisciplinary model that integrates metabolic assessment, feeding capacity, and longitudinal growth monitoring into a cohesive and adaptive care pathway. Optimization of oral protein intake cannot be achieved through isolated interventions; rather, it requires coordinated input from pediatric cardiologists, dietitians, feeding specialists, nurses, and caregivers. This integrated model enables comprehensive evaluation of cardiac status, energy expenditure, oral motor function, and dietary intake, allowing clinicians to tailor protein delivery strategies to the child’s dynamic clinical condition. The recent pediatric nutrition and cardiac care literature suggests that multidisciplinary nutrition support teams are associated with improved attainment of protein and energy targets, better growth outcomes, and reduced postoperative complications in medically complex children [86,87]. In CHD specifically, coordinated nutritional care has been associated with more favorable weight trajectories and may help reduce growth faltering when interventions are initiated early and continuously adapted [2].

Furthermore, integrating nutritional care into routine clinical management ensures that feeding strategies remain responsive to the fluctuating metabolic demands characteristic of CHD, particularly in the perioperative period. Children with CHD often transition rapidly between catabolic and anabolic states, requiring frequent recalibration of protein and energy targets [9]. Multidisciplinary teams facilitate this dynamic process through regular growth assessment, dietary monitoring, and clinical evaluation, enabling timely modifications to feeding plans. Such a feedback-driven approach is essential to maintain protein adequacy and prevent cumulative deficits that contribute to growth failure. Longitudinal studies have shown that sustained, team-based nutritional management is associated with improved growth trajectories and may mitigate the risk of neurodevelopmental impairment linked to early-life undernutrition in CHD patients [88]. Additionally, coordinated care facilitates caregiver engagement and education, ensuring that oral feeding strategies are effectively translated from the hospital to the home environment, thereby improving adherence and sustainability [17,89]. The evidence synthesized across the preceding sections demonstrates that oral protein optimization in children with CHD requires addressing both their increased metabolic demands and various feeding-related barriers. To facilitate interpretation of the available evidence, representative studies and their principal findings are summarized according to the major thematic domains discussed in this review in Table 2.

## 6. Practical Clinical Framework for Nutritional Management

The available evidence supports a stepwise and individualized approach to optimizing oral protein intake in children with congenital heart disease (CHD). Rather than applying a uniform nutritional prescription, the proposed framework integrates nutritional screening, assessment of feeding barriers, individualized oral feeding strategies, regular reassessment, and escalation of nutritional support when oral intake remains insufficient. This approach is intended to translate the evidence summarized in the preceding sections into practical clinical decision-making while recognizing the heterogeneity of children with CHD and the limited availability of CHD-specific intervention studies [23,90].

First, early nutritional screening and growth assessment should be incorporated into routine clinical care. Assessment may include body weight, length or height, head circumference in infants, growth velocity, and longitudinal changes in weight-for-age, weight-for-length/height, and length/height-for-age z-scores [32,91]. Serial measurements are more informative than isolated anthropometric values because they allow clinicians to identify persistent growth faltering and evaluate the response to nutritional intervention. The frequency of monitoring can be adjusted according to nutritional risk, disease severity, and clinical stability, as summarized in Table 1 [92].

Second, the causes of inadequate oral protein intake should be identified before selecting an intervention. A structured feeding assessment should consider feeding endurance, suck–swallow–breathe coordination, feeding duration, gastrointestinal symptoms, early satiety, oral motor function, and caregiver-related factors [46,75,81]. Identifying the predominant limitation is important because the appropriate intervention may differ according to whether inadequate intake is primarily related to feeding fatigue, restricted feeding volume, swallowing dysfunction, gastrointestinal intolerance, or other clinical factors. This assessment also provides a basis for determining whether oral feeding remains feasible and which strategies are most appropriate for the individual child [93].

Third, oral protein delivery can be optimized according to the identified feeding limitations. When oral feeding remains feasible, but intake is insufficient, increasing nutrient density through energy- and protein-dense foods or formulas, food fortification, or ONS may be considered. Smaller and more frequent meals may also be used when feeding endurance is limited. These strategies should be individualized according to age, nutritional status, feeding tolerance, cardiac condition, and dietary preferences [23,46,60,71,85,91]. Rather than assuming that a single intervention is appropriate for all children, the choice and combination of strategies should be guided by the child’s specific nutritional and feeding profile.

Fourth, the response to nutritional intervention should be reassessed systematically. Follow-up should incorporate serial anthropometric measurements together with evaluation of dietary intake, feeding performance, tolerance, and achievement of individualized nutritional targets [32,34,38,94]. Lack of improvement in growth trajectory, persistent inadequacy of protein or energy intake, or worsening feeding difficulties should prompt reassessment of the underlying barriers and modification of the nutritional plan. This iterative process allows nutritional management to be adjusted as the child’s cardiac status, feeding capacity, and nutritional requirements change [23].

Finally, escalation of nutritional support may be considered when optimized oral strategies do not provide adequate intake or satisfactory growth. Supplemental tube feeding can be used as an adjunct to oral feeding when clinically appropriate, while opportunities for oral stimulation and developmentally appropriate oral feeding should be maintained whenever feasible [23,38,95]. The decision to escalate should take into account the child’s growth trajectory, nutritional intake, feeding tolerance, cardiac status, and risk associated with inadequate intake. A multidisciplinary team involving pediatric cardiologists, dietitians, feeding specialists, nurses, and caregivers may facilitate coordinated assessment and individualized implementation of the nutritional plan [57].

The proposed framework therefore emphasizes a sequential approach comprising nutritional screening, identification of feeding barriers, individualized oral protein optimization, regular reassessment, and escalation of nutritional support, when necessary, rather than prescribing a fixed protein-delivery strategy for all children with CHD. Figure 2 summarizes this clinical pathway. Given the limited CHD-specific evidence and the substantial contribution of observational studies, expert consensus, and evidence extrapolated from other pediatric populations, the framework should be regarded as a practical synthesis of the available evidence rather than a definitive clinical guideline [23,52].

## 7. Clinical Outcomes, Evidence Gaps, and Future Directions

### 7.1. Short-Term Clinical Outcomes

Available evidence suggests that nutritional adequacy is associated with selected short- and long-term clinical outcomes in children with CHD. Adequate protein provision may support tissue repair, immune function, and recovery during periods of postoperative metabolic stress [96,97]. Protein inadequacy may contribute to the catabolic response, impair tissue repair, and increase susceptibility to infection, ultimately contributing to prolonged hospitalization and higher morbidity [58]. Conversely, studies have demonstrated that achieving nutritional targets in the early postoperative phase is associated with a shorter intensive care unit stay, reduced complication rates, and improved clinical stabilization [98].

From a health systems perspective, improving oral protein intake also has implications for reducing healthcare burden. Children who achieve adequate nutritional status tend to have shorter hospital stays, fewer readmissions, and reduced need for intensive nutritional interventions, thus highlighting the role of nutrition as a modifiable factor in improving both patient outcomes and healthcare efficiency [99,100]. Furthermore, the long-term impacts of early protein adequacy on neurodevelopmental outcomes require further investigation. Addressing these gaps will be essential to strengthen the evidence base and inform clinical guidelines.

### 7.2. Growth and Neurodevelopment

Beyond immediate recovery, protein intake plays a central role in determining growth and body composition. Children with CHD are at high risk of growth faltering due to chronic energy imbalance and increased metabolic demands, with prevalence rates of undernutrition exceeding 25% globally in this population [58]. Adequate protein intake may support lean body mass accretion and contribute to catch-up growth when other nutritional and clinical barriers are addressed. Longitudinal cohort studies have demonstrated that improved nutritional status is associated with better growth trajectories and reduced severity of malnutrition in this population. This is clinically significant, as growth failure in CHD has been consistently linked to adverse neurodevelopmental outcomes and reduced functional capacity later in life [2,58].

Early nutritional adequacy has been associated with more favorable developmental outcomes, although direct evidence linking oral protein optimization with neurodevelopment remains limited. These outcomes are likely mediated through the impacts of protein on brain development, synaptic formation, and overall somatic growth. Emerging evidence has further suggested that optimizing early nutrition may attenuate the long-term impacts of chronic inflammation and catabolism on growth and development [101].

### 7.3. Limitations of Current Evidence

Despite increasing recognition of the importance of protein intake in children with CHD, the current evidence base remains limited. Much of the available literature has focused on overall nutritional support, perioperative nutrition, or energy provision, rather than oral protein optimization as a distinct therapeutic target. High-quality randomized controlled trials evaluating oral protein-focused interventions remain scarce, and considerable heterogeneity exists in the reported study populations, disease severity, nutritional assessment methods, and clinical outcome measures. This variability limits direct comparison across studies and hinders the development of standardized clinical recommendations.

Another important limitation is that many existing recommendations are extrapolated from studies involving critically ill children or those with other pediatric chronic diseases, as CHD-specific evidence remains insufficient. Although these findings provide valuable mechanistic insights, they may not fully reflect the unique metabolic demands, feeding difficulties, and growth patterns observed in children with CHD.

### 7.4. Research Priorities

Future research should focus on establishing evidence-based oral protein recommendations tailored to the clinical characteristics and nutritional risks of children with CHD. Well-designed multicenter randomized controlled trials are needed to determine optimal protein intake levels according to age, disease severity, and perioperative status, while simultaneously evaluating clinically meaningful outcomes such as growth recovery, lean body mass, postoperative complications, feeding tolerance, neurodevelopment, and quality of life.

Additional studies should also investigate the timing of the nutritional intervention, the comparative effectiveness of different oral protein delivery strategies, and the integration of individualized nutritional management strategies within multidisciplinary care pathways. Strengthening the evidence in these areas can be expected to facilitate the development of standardized clinical guidelines and support more consistent implementation of protein-focused nutritional care in the pediatric CHD population.

## 8. Conclusions

Children with congenital heart disease have increased protein requirements that are frequently difficult to meet through oral feeding due to hypermetabolism, feeding fatigue, gastrointestinal dysfunction, oral motor impairment, and other disease-related challenges. This narrative review synthesized the currently available evidence and highlights the potential value of individualized, protein-focused nutritional strategies to address both increased metabolic demands and feeding-related limitations. Available evidence suggests that nutrient-dense feeding, food fortification, oral nutritional supplementation when appropriate, and multidisciplinary feeding support may help improve protein delivery and nutritional adequacy. As children with congenital heart disease represent a clinically heterogeneous population, nutritional management should be individualized according to the patient’s age, underlying cardiac lesion, physiological status, and feeding capacity. As current recommendations remain limited by the predominance of observational studies, expert consensus, and evidence extrapolated from other pediatric populations, well-designed prospective multicenter studies and randomized clinical trials specifically involving children with congenital heart disease are needed in order to establish standardized, evidence-based recommendations for oral protein management across different ages and clinical conditions.

## Figures and Tables

**Table 1:** Clinical growth monitoring recommendations for children with congenital heart disease.

Parameter	Practical Target	Clinical Considerations	Suggested References
Body weight	Maintain or improve weight-for-age (WAZ) or weight-for-length/height z-score along the expected growth trajectory.	Failure to regain the expected growth trajectory or persistent decline in WAZ should prompt nutritional reassessment.	WHO Child Growth Standards (2006) [32]; Mehta et al. (2013) [33]
Length/Height	Maintain age-appropriate linear growth and improve LAZ/HAZ over time.	Persistent linear growth faltering may indicate chronic nutritional inadequacy or unresolved disease burden.	WHO Child Growth Standards (2006) [32]
Head circumference	Maintain head circumference along age- and sex-specific percentiles during the first two years of life.	Slowing HC growth may indicate inadequate nutrition or impaired neurodevelopment.	WHO Child Growth Standards (2006) [32]
Growth velocity	Growth velocity should meet or exceed the expected rate for age; accelerated growth is expected during nutritional rehabilitation.	Serial measurements are more informative than isolated measurements.	WHO Child Growth Standards (2006) [32]
Growth status	Inadequate growth, growth maintenance, catch-up growth.	Based on serial anthropometric assessment.	WHO Child Growth Standards (2006) [32]
Monitoring frequency	Every 2–4 weeks in nutritionally vulnerable infants; every 1–3 months in clinically stable children.	Individualize according to nutritional risk and disease severity.	Puntis et al. (2018) [34]; Mehta et al. (2009) [35]
Growth reference	WHO Child Growth Standards; syndrome-specific growth charts when appropriate.	Down, Turner, Noonan syndrome, etc.	WHO Child Growth Standards (2006) [32]; Zemel et al. (2015) [31]; Lyon et al. (1985) [36]
Escalation to supplemental tube feeding	Consider tube feeding when oral intake remains inadequate despite individualized oral feeding strategies.	Consider growth trajectory, feeding tolerance, and cardiac status.	Lichtenstein et al. (2021) [37]; Zemel et al. (2015) [31]; Braegger et al. (2010) [38]; Mehta et al. (2013) [33]; Mehta et al. (2009) [35]

**Table 2:** Summary of representative studies and principal findings supporting oral protein optimization in children with congenital heart disease.

Domain	Representative Evidence	Study Design	Main Findings	Clinical Implications
Increased protein and energy requirements	[2,9,21,23]	Narrative review, consensus statement, review	Children with CHD exhibit increased energy expenditure and accelerated protein turnover due to hypermetabolism, chronic inflammation, heart failure, and perioperative stress. Protein requirements may increase to 3–4 g/kg/day in high-risk patients.	Nutritional management should prioritize adequate protein provision, rather than caloric intake alone, to support growth, immune function, and postoperative recovery.
Growth failure and nutritional vulnerability	[3,4,28,58]	Systematic review, meta-analysis, cohort study	Growth faltering, underweight, wasting, and stunting are highly prevalent in children with CHD, particularly among infants with complex cardiac lesions. Poor nutritional status is associated with adverse clinical outcomes.	Early nutritional screening and serial anthropometric monitoring are essential to identify nutritional deterioration and guide intervention.
Feeding fatigue and cardiorespiratory limitation	[17,39,46]	Scoping review, review	Increased work of breathing, reduced feeding endurance, fatigue, and prolonged feeding duration substantially reduce oral intake and protein delivery.	Smaller, more frequent meals and individualized feeding schedules may improve feeding efficiency while minimizing cardiorespiratory stress.
Gastrointestinal dysfunction and early satiety	[10,25,42]	Narrative reviews	Gastrointestinal hypoperfusion, delayed gastric emptying, gastroesophageal reflux, and protein-losing enteropathy reduce feeding capacity and impair protein absorption.	Management should combine treatment of gastrointestinal symptoms with nutrient-dense oral feeding strategies to maximize protein intake.
Oral motor dysfunction and swallowing impairment	[11,44,45,46]	Cohort study, review	Oral motor dysfunction, dysphagia, aspiration risk, and impaired suck–swallow–breathe coordination frequently delay achievement of full oral feeding in infants, particularly after cardiac surgery.	Early multidisciplinary feeding assessment involving speech-language therapists, occupational therapists, and dietitians is recommended to preserve oral feeding skills.
Energy- and protein-dense feeding	[52,59,61]	Systematic review, randomized trial, review	Increasing nutrient density improves protein adequacy, weight gain, nitrogen balance, and postoperative recovery without increasing feeding volume.	Protein-dense feeding should be considered a first-line oral nutritional strategy for children with limited feeding capacity.
Food fortification	[60,61]	Cohort study, randomized trial	Fortification of milk or habitual foods increases protein and energy intake within tolerated feeding volumes and improves growth outcomes.	Food fortification provides a practical and cost-effective approach for outpatient and inpatient nutritional management.
Oral nutritional supplementation (ONS)	[2,61]	Randomized trial, review	ONS improves total protein intake, weight gain, and nutritional status, particularly among children unable to meet requirements through habitual diets alone.	ONS should complement, rather than replace, individualized oral feeding plans.
Supplemental tube feeding	[23,38,46]	Review, guideline, consensus	Supplemental enteral feeding is indicated when oral strategies fail to achieve adequate intake or satisfactory growth, while oral feeding skills should be maintained whenever feasible.	Tube feeding should be considered an adjunct to oral feeding within individualized nutritional management.
Multidisciplinary nutritional care	[9,17,25]	Narrative reviews	Coordinated care involving pediatric cardiologists, dietitians, speech-language therapists, nurses, and caregivers improves nutritional assessment, feeding support, and long-term growth outcomes.	Multidisciplinary nutritional care should be integrated into routine management throughout the disease course.
Evidence gaps and future priorities	Current review synthesis	Narrative synthesis	High-quality CHD-specific intervention studies evaluating oral protein optimization remain limited. Considerable heterogeneity exists in age groups, disease severity, nutritional assessment methods, and outcome measures.	Well-designed prospective multicenter studies are needed to establish standardized protein recommendations and oral feeding protocols across different pediatric age groups and clinical settings.

## Data Availability

No new data were created or analyzed in this study. This article is a narrative review based on previously published literature, and data sharing is not applicable to this article.

## Abbreviations

The following abbreviations are used in this manuscript:

CHD	Congenital Heart Disease
ICU	Intensive Care Unit
PRISMA	Preferred Reporting Items for Systematic Reviews and Meta-Analyses
HF	Heart Failure
PICU	Pediatric Intensive Care Unit
REE	Resting Energy Expenditure
PLE	Protein-losing Enteropathy
VFMI	Vocal Fold Motion Impairment
ONS	Oral Nutritional Supplementation
